# Universal activation function for machine learning

**DOI:** 10.1038/s41598-021-96723-8

**Published:** 2021-09-21

**Authors:** Brosnan Yuen, Minh Tu Hoang, Xiaodai Dong, Tao Lu

**Affiliations:** Department of Electrical and Computer Engineering, University of Victoria, Victoria, BC Canada

**Keywords:** Computational science, Scientific data, Information technology, Electrical and electronic engineering

## Abstract

This article proposes a universal activation function (UAF) that achieves near optimal performance in quantification, classification, and reinforcement learning (RL) problems. For any given problem, the gradient descent algorithms are able to evolve the UAF to a suitable activation function by tuning the UAF’s parameters. For the CIFAR-10 classification using the VGG-8 neural network, the UAF converges to the Mish like activation function, which has near optimal performance $F_{1}=0.902\pm 0.004$ when compared to other activation functions. In the graph convolutional neural network on the CORA dataset, the UAF evolves to the identity function and obtains $F_1=0.835\pm 0.008$. For the quantification of simulated 9-gas mixtures in 30 dB signal-to-noise ratio (SNR) environments, the UAF converges to the identity function, which has near optimal root mean square error of $0.489\pm 0.003~\mu {\mathrm{M}}$. In the ZINC molecular solubility quantification using graph neural networks, the UAF morphs to a LeakyReLU/Sigmoid hybrid and achieves RMSE=$0.47\pm 0.04$. For the BipedalWalker-v2 RL dataset, the UAF achieves the 250 reward in ${961\pm 193}$ epochs with a brand new activation function, which gives the fastest convergence rate among the activation functions.

## Introduction

The goal of most machine learning algorithms is to find the optimal model for a specific problem. However, finding the optimal model by hand is a daunting task due to the virtually infinite number of possibilities on model and the corresponding parameter selection. The field of automated machine learning^1–3^ solves the problem by automatically finding machine learning models using genetic algorithms, neural networks and its combination with probabilistic and clustering algorithms.

Genetic algorithms excel at optimizing discrete variables. For example, they can be used to optimize the number of neurons in each layer or the depth of the neural network. Neuroevolution of augmenting topologies (NEAT)^4^ uses genetic algorithms to optimize the structure of neural networks. The values of the neuron weights, the types of activation functions, and the number of neurons can be optimized by breeding and mutating different species of neural networks. HyperNEAT^5^ is an extension of NEAT. Instead of finding the architecture directly, HyperNEAT finds a single function that encodes the entire network. The single function is then bred and mutated in order to find the best function that encodes the optimal neural architecture. Moreover, Deep HyperNEAT^6^ is another version of HyperNEAT that allows the design of larger and deeper neural networks.

Aside from the genetic algorithms, neural network structures can also be optimized by other neural networks. Liu et al.^7^ propose a new method for creating convolutional neural networks (CNNs) from scratch. CNNs are constructed from cells, where each cell does a specific operation such as convolution, concatenation, and pooling. Moreover, the cells come with fixed activation functions that format the outputs. A neural network predictor is trained to place and route cells together. The architecture begins as a collection of a few cells and more cells are added by the predictor until the lowest loss is achieved. Similar to the paper above, Efficient Neural Architecture Search via Parameter Sharing (ENAS)^8^ uses a recurrent neural network (RNN) controller to place and route cell blocks in order to find the optimal architecture. The RNN controller is trained using the policy gradient method. On the other hand, the Auto-DeepLab paper^9^ proposes a method to search architectures at a cell level and at the network level.

Probabilistic methods could be used in-conjunction with neural network approaches to create new neural network architectures. Zoph et al.^10^ designed an RNN controller for neural architecture search, which is trained using reinforcement learning. The RNN controller searches through the vast array of possible neural networks and it labels each network with a probability of being the optimal network. Moreover, it predicts the optimal parameters of the neural network such as the size of the CNN filters, the number of CNN channels, and the types of activation functions.

Clustering algorithms can be used to find the type of problem based on the information from the dataset. For example, the problem may be classified as a video quantification problem or a text classification problem or a reinforcement learning problem. Subsequently, the best neural network is selected from a pre-built model zoo and it is retrained to get the best results.

One of the core tasks for automated machine learning is to find an optimal activation function for a specific model. However, many activation functions have been proposed over the history of machine learning and this makes the selection difficult. Richards^11^ developed the sigmoid activation function family that spans the S-shaped curves like the tanh^12^ function and the sigmoid function. Other activation functions in the family include the step function, the clipped tanh function, and the clipped sigmoid function. Subsequently, the first neural network^13,14^ used the sigmoid activation function for modeling biological neuron firing. For the most part, activation functions from the sigmoid activation function family are used for classifying objects, where the output is constrained to the range [0, 1].

The ReLU activation function^15^ is another popular activation function that is used for quantification, classification, and reinforcement learning problems. The ReLU activation function is part of the ReLU activation function family, where the behaviour of all functions in the family are linear $y=x$ when $x>0$. The identity, LeakyReLU^16^, Elu^17^, and softplus^18^ activation functions are also included in this family. The LeakyReLU activation function is a version of ReLU activation function that has a non-zero slope $y=\alpha x$ when $x<0$, where the non-zero slope is used to prevent the gradient from reaching zero. One of the major problems of the ReLU and LeakyReLU activation functions is the discontinuity at $x=0$ that produces undefined gradients^19^ and causes the gradient descent optimizer to fail. The Elu and the softplus activation functions solve the problem by creating smoothness and continuity around $x=0$^18^. Newer activation functions such as Mish^20^ and Swish^21^ have built-in regularization to prevent over-fitting of models.

The Gaussian activation function^22^ has a bell shaped curve and it is useful for modeling Gaussian distributed random variables. For example, a neural network predicting the speed of a car might use the Gaussian function for regression because the speed of a car is Gaussian distributed^23^. Moreover, the Gaussian function is also used for classifying the existence of objects^24^. The Gaussian function is a special case of the radial basis function (RBF) activation function family^24^, whose functions always have a bell shape curve. Other members of the RBF family include the polyharmonic spline and the bump function.

Among the many basic activation functions, selecting the best activation function that suits a specific task is hard. Researchers have solved this problem by creating adaptable activation functions that can evolve to a specific task. The adaptable activation functions are controlled by trainable parameters, of which are then optimized using gradient descent algorithms. PReLU^25^ is an example of an adaptive activation function, where the slope $\alpha$ of a LeakyReLU function is a trainable parameter. Bodyanskiy et al.^26^ developed an adaptable RBF that can be trained in real time. Qian et al.^27^ proposed adaptive ReLU functions for CNNs. Campolucci et al.^28^ used an adaptive spline activation function that approximates the curves of a sigmoid activation function. However, the adaptive spline activation functions suffer from over-fitting and discontinuities. Each individual spline is constructed independently of other segments. Afterwards, the segments are joined together to form a complete activation function. As a result, continuity is not guaranteed at the segment joints because the derivatives of the two different segments might not agree. Furthermore, too many segments might introduce a large amount of trainable parameters and this might cause over-fitting^29^.

We propose a simple universal activation function (UAF) to solve the problem of finding the optimal activation function for a specific task. The 5 trainable parameters of the UAF allows it to approximate any of the activation functions listed above. Without any additional constraints, the UAF is continuous and differentiable for all parameter values. Due to the properties above, the gradient descent algorithms are able to smoothly evolve the UAF to a near optimal activation function, which may be an existing activation function in the literature or a new activation function. Adopting the UAF in neural networks automates the search for a good activation function and reduces the total training time. For example, NEAT^4^ and ENAS^8^ discretely search through the activation functions one by one. Everytime the activation function changes, the neural networks above need to be retrained from scratch. Instead of retraining the neural networks, the activation functions and the weights can be continuously updated to reduce training time. These papers^30,31^ prove that adaptive activation functions converge faster for certain problems such as stiff ordinary differential equations and partial differential equations.

The paper is organized as follows. Section “Construction of UAF” describes the properties of UAF and its training procedure. Section “Experiments” shows the UAF’s performances on the CIFAR-10^32^ classification, infrared spectra database for 9 gas quantification^33^, BipedalWalker-v2^34^ RL, Planetoid/CORA publication classification dataset^35^, and ZINC molecular solubility quantification dataset^36^. Furthermore, a conclusion is presented in Section “Conclusion and future work”. Finally, Supplementary Information S.2 gives implementation details about the UAF.

## Construction of UAF

In this section, the UAF will be derived from the softplus activation function. For the range of $x\gg 0$, the ReLU activation function can be approximated by the softplus activation function.

1 $$\begin{aligned} softplus(x)= \ln (1+e^{x}) \approx ReLU(x) \end{aligned}$$

Furthermore, the softplus function can be generalized by adding two new trainable parameters *A* and *B*

2 $$\begin{aligned} f_{UAF}(x) =\ln (1+e^{A(x+B)}) \end{aligned}$$

where *A* controls the slope and *B* controls the horizontal shift. The LeakyReLU activation function can be approximated by adding another monotonically decreasing function and a new parameter *D*

3 $$\begin{aligned} f_{UAF}(x) =\ln (1+e^{A(x+B)}) - \ln (1+e^{D(x-B)} ) \end{aligned}$$

that approximates the slope $\alpha$ of the LeakyReLU activation function. Moreover, the sigmoid and tanh activation functions can be approximated by adding a new parameter *E*

4 $$\begin{aligned} f_{UAF}(x) =\ln (1+e^{A(x+B)}) - \ln (1+e^{D(x-B)} ) + E \end{aligned}$$

that controls the vertical shift. The sigmoid activation function can be transformed into the tanh activation function by shifting the function down by *E*. In order to approximate the Gaussian activation function, a new parameter *C* is added

5 $$\begin{aligned} f_{UAF}(x) =\ln (1+e^{A(x+B)+Cx^{2}}) - \ln (1+e^{D(x-B)} ) + E \end{aligned}$$

to give more degrees of freedom to the UAF. The completed $f_{UAF}(x)$ is shown in Eq. (5). In the Supplementary Materials, there is a video (V.1 describing the effects of the parameters on the UAF. It is evident that the UAF given by Eq. (5) is well behaved such that both the function and its first order derivative exist, are single valued and continuous for $x \in (-\infty ,\infty )$ provided that all parameters are real.

**Figure 1:**
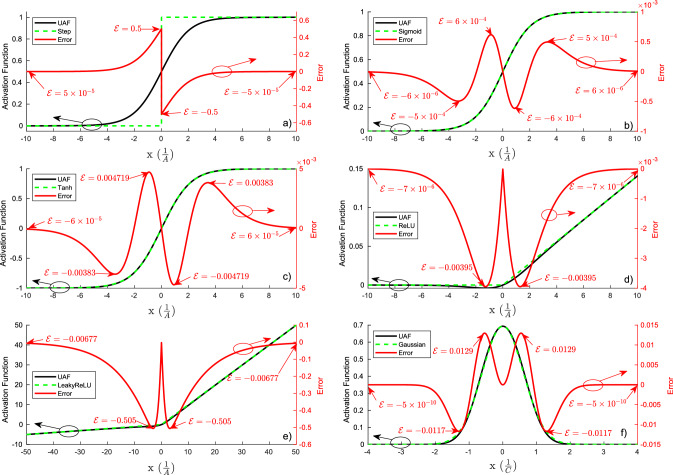
The UAF’s approximations of the following activation functions: (**a**) step, (**b**) sigmoid, (**c**) tanh, (**d**) ReLU, (**e**) LeakyReLU, and (**f**) Gaussian. The black solid lines represent the UAF, while the green dashed lines represent the targeted activation functions, whose values can be obtained from the *y* axis on the left. The red solid lines represent the error $\mathcal{E}$ between the UAF and targeted activation function and the values can be read from the *y* axis on the right side.

### UAF error analysis using RMSE table

In this subsection, we will examine the errors of the UAF in the range of $x \in [-5,5]$ because every maximum absolute error occurs within this range. Table 1 shows the root mean square error (RMSE), mean absolute error (MAE), maximum absolute error, and locations of the maximum absolute error for each activation function. The UAF models the identity function and the softplus function without any errors because the UAF is based on those functions. For the continuous activation functions such as the sigmoid, tanh, and Gaussian, the UAF models them well with a small RMSE. For the discontinuous activation functions like the ReLU and LeakyReLU, the RMSE is slightly higher due to the continuous UAF not being able to handle the discontinuities. A more through evaluation of the UAF’s error analysis is available in the Supplementary Information S.1.

**Table 1:** UAF approximation errors of various activation functions.

Activation functions	RMSE	MAE	Max abs. err.	Locations of max abs. err.
Identity	0	0	0	None
Step	$1.2\times 10^{-2}$	$5.0\times 10^{-4}$	0.5	$0^{\pm }$
ReLU	$7.5\times 10^{-5}$	$4.1\times 10^{-6}$	$4.0\times 10^{-3}$	$\pm 0.0181$
LeakyReLU	$6.2\times 10^{-2}$	$3.6\times 10{-2}$	0.505	$\pm 3.106$
Sigmoid	$3.0\times 10^{-4}$	$2.0\times 10^{-4}$	$6.0\times 10^{-4}$	$\pm 0.8665$
Tanh	$1.5\times 10^{-3}$	$8.0\times 10^{-4}$	$4.7\times 10^{-3}$	$\pm 0.4355$
Softplus	0	0	0	None
Gaussian	$4.6\times 10^{-3}$	$2.4\times 10^{-3}$	$1.3\times 10^{-2}$	$\pm 0.8821$

### UAF error analysis using error plots

To further illustrate the errors between the UAF and the targeted activation functions, we have made error plots of the UAF as shown in Fig. 1. The UAF (black solid traces) can closely approximate various activation functions (green dashed traces) such as step (Fig. 1a), sigmoid (Fig. 1b), tanh (Fig. 1c), ReLU (Fig. 1d), LeakyReLU (Fig. 1e) and Gaussian (Fig. 1f) with red traces showing monotonically decreasing errors toward $\pm \,  \infty$. Details on UAF’s parameter values in each approximation and the corresponding error analysis are described in Supplementary Information S.1.

## Training the UAF’s parameters

Unlike regular activation functions, the UAF has trainable parameters and it requires a unique training procedure to achieve the best performance. The exact same training procedure is followed for each dataset in “Experiments”. The UAF’s training procedure is divided into phase 1 and phase 2. Starting with training phase 1, gradients of the weights, biases, and UAF’s parameters are computed. Afterwards, the ADAM optimizer^37^ updates the weights, biases, and UAF’s parameters concurrently using the computed gradients. When the loss function hits a plateau, training phase 1 ends and training phase 2 begins.

In training phase 2, the ADAM optimizer only updates the weights and biases of the neural network, while the UAF’s parameters are not updated. This is done to reduce the over-fitting of the model and to prevent training instability. In order to update the UAF’s parameters, the ADAM optimizer requires the UAF’s gradients. Derivation of the UAF’s gradients is presented below.

### Derivation of the UAF’s gradients

Suppose a MSE loss function *J* needs to be minimized

6 $$\begin{aligned} J = ({\hat{y}}-y)^2 \end{aligned}$$

by tuning the predicted output ${\hat{y}}$ to match the actual output *y*. Suppose the predicted output ${\hat{y}}$ is modeled by a single layer MLP that has the UAF

7a $$\begin{aligned}&x = v + \sum ^N_{i=1} w_i x_i \end{aligned}$$

7b $$\begin{aligned}&{\hat{y}} = f_{UAF} \left( x ,A,B,C,D,E \right) \end{aligned}$$

where $x_i$ are the inputs, $w_i$ are the weights, and *v* is the bias. Firstly, the UAF’s gradients $\nabla f_{UAF}(x,A,B,C,D,E)$

8a $$\begin{aligned} \dfrac{ \partial f_{UAF}}{ \partial x}&= \dfrac{ (A+2Cx)e^{A(x+B)+Cx^2}}{ 1+e^{A(x+B)+Cx^2}} - \dfrac{ De^{D(x-B)}}{ 1+e^{D(x-B)}} \end{aligned}$$

8b $$\begin{aligned} \dfrac{ \partial f_{UAF}}{ \partial A}&=\dfrac{ (x+B)e^{A(x+B)+Cx^2}}{ 1+e^{A(x+B)+Cx^2}} \end{aligned}$$

8c $$\begin{aligned} \dfrac{ \partial f_{UAF}}{ \partial B}&=\dfrac{ Ae^{A(x+B)+Cx^2}}{ 1+e^{A(x+B)+Cx^2}} \end{aligned}$$

8d $$\begin{aligned} \dfrac{ \partial f_{UAF}}{ \partial C}&=\dfrac{ x^2 e^{A(x+B)+Cx^2}}{ 1+e^{A(x+B)+Cx^2}} \end{aligned}$$

8e $$\begin{aligned} \dfrac{ \partial f_{UAF}}{ \partial D}&=- \dfrac{ (x-B)e^{D(x-B)}}{ 1+e^{D(x-B)}} \end{aligned}$$

8f $$\begin{aligned} \dfrac{ \partial f_{UAF}}{ \partial E}&= 1 \end{aligned}$$

are computed. Secondly, the loss function’s gradients $\nabla J$

9a $$\begin{aligned}&\nabla J = 2({\hat{y}}-y ) \nabla f_{UAF}(x,A,B,C,D,E) \end{aligned}$$

9b $$\begin{aligned}&\dfrac{ \partial J}{ \partial w_i} = 2({\hat{y}}-y ) \dfrac{ \partial f_{UAF}(x,A,B,C,D,E)}{ \partial x} x_i \end{aligned}$$

are calculated. Thirdly, the UAF’s parameters

10a $$\begin{aligned}&m(t) = \beta _1 m(t-1) + (1-\beta _1 ) \nabla J(t) \end{aligned}$$

10b $$\begin{aligned}&h(t) = \beta _2 h(t-1) + (1-\beta _2 ) \left( \nabla J(t) \right) ^2 \end{aligned}$$

10c $$\begin{aligned}&{\hat{m}}(t) = \frac{m(t)}{1-\beta _1 } \end{aligned}$$

10d $$\begin{aligned}&{\hat{h}}(t) = \frac{h(t)}{1-\beta _2 } \end{aligned}$$

10e $$\begin{bmatrix} v(t + 1)\\ A(t + 1)\\ B(t + 1)\\ C(t + 1)\\ D(t + 1)\\ E(t + 1) \end{bmatrix} = \begin{bmatrix} v(t)\\ A(t)\\ B(t)\\ C(t)\\ D(t)\\ E(t) \end{bmatrix} - \alpha (t) \frac{{\hat{m}}(t)}{\sqrt{{\hat{h}}(t) + \epsilon }}$$

are updated using the ADAM optimizer. The ADAM optimizer also requires the learning rates, which are described in the next subsection.

### Learning rates for phase 1 and phase 2

In training phase 1, the learning rate is held constant $\alpha (t) = \alpha _0$ for epochs $0< t < t_0$. When the loss does not decrease for *Z* epochs, the loss is considered to have plateaued at epoch $t_0$ and this leads to the start of training phase 2. In training phase 2, the new learning rate $\alpha (t) = \alpha _1$ is significantly smaller than the previous learning rate $\alpha _1 < \alpha _0$. Moreover, the learning rate decreases when the loss has plateaued for *Z* epochs.

**Figure 2:**
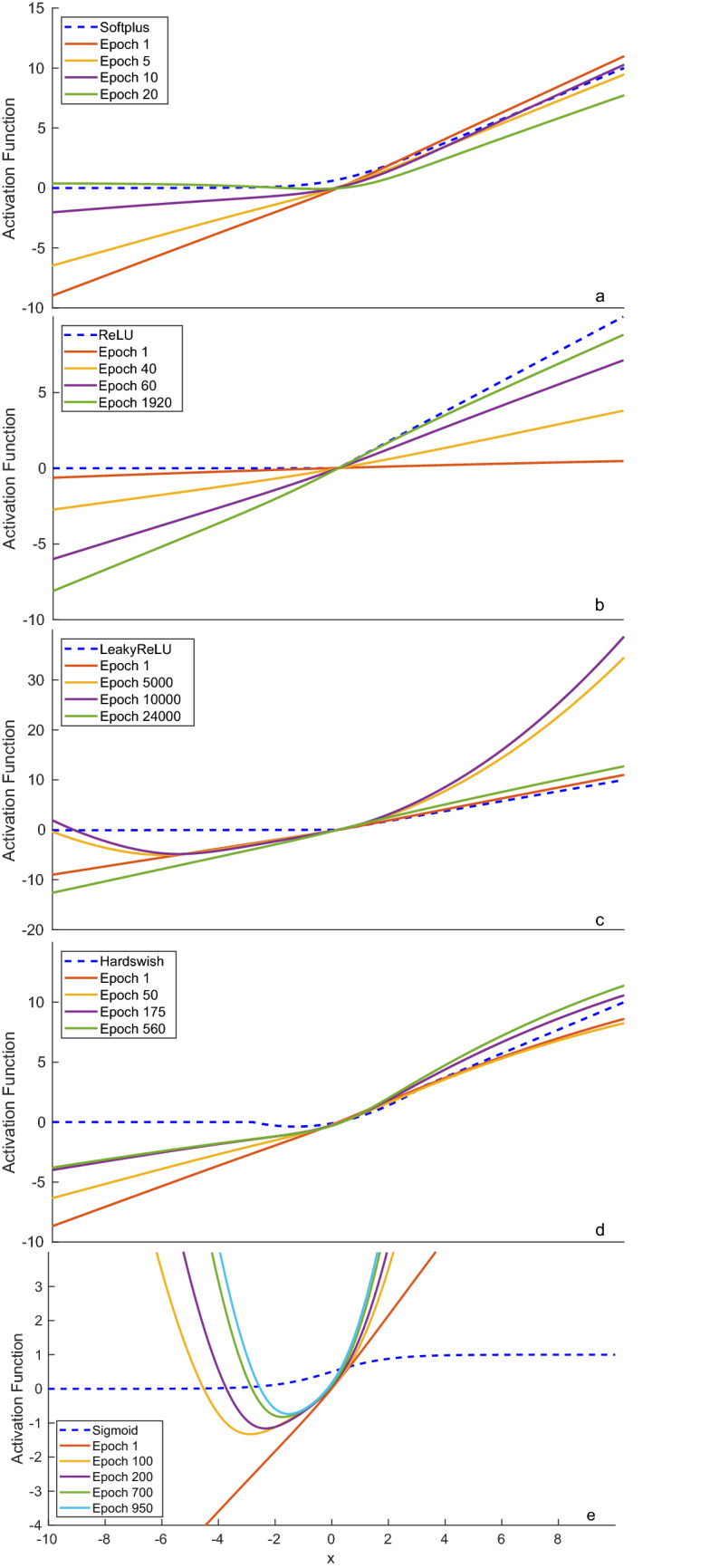
The UAF evolution of the following datasets: (**a**) CIFAR-10 image classification, (**b**) CORA publication classification, (**c**) 9 gas concentration quantification, (**d**) ZINC molecular solubility quantification, and (**e**) BipedalWalker-V2 reinforcement learning.

## Experiments

In this article, five experiments are used to benchmark the UAF against other activation functions. To show the effectiveness of the UAF, an animation depicting the evolution of the UAF in these datasets is available in the Supplementary Materials (V.2).

### CIFAR-10 image classification

The goal of the CIFAR-10 dataset^32^ is to take $32 \times 32$ pixel RGB images and classify them into 10 different categories: airplane, automobile, bird, cat, deer, dog, frog, horse, ship, and truck. The VGG 8 layer CNN^38^ is applied to the CIFAR-10 dataset, of which contains 6 CNN layers, 6 max pooling layers, and 2 dense layers. Each CNN layer contains many $3 \times 3$ pixel kernels interspersed with max pooling layers. On the other hand, each dense layer has 1,024 neurons and they produce the output classification result.

**Table 2:** CIFAR-10 image classification using VGG 8 layers.

Activation functions	Precision	Recall	$\mathrm{F}_{1}$	Training time ($\times 10^3$s)
Softplus	$0.902 \pm 0.003$	$0.902 \pm 0.003$	$0.902 \pm 0.003$	$0.699 \pm 0.002$
**UAF**	$0.902 \pm 0.004$	$0.902 \pm 0.004$	$0.902 \pm 0.004$	$1.692 \pm 0.004$
LeakyReLU	$0.893 \pm 0.003$	$0.893 \pm 0.003$	$0.893 \pm 0.003$	$0.863 \pm 0.005$
Mish	$0.891 \pm 0.008$	$0.890 \pm 0.008$	$0.891 \pm 0.008$	$0.927 \pm 0.005$
ELU	$0.886 \pm 0.004$	$0.886 \pm 0.004$	$0.886 \pm 0.004$	$0.699 \pm 0.003$
Sigmoid	$0.882 \pm 0.004$	$0.881 \pm 0.006$	$0.881 \pm 0.006$	$0.686 \pm 0.010$
Tanh	$0.839 \pm 0.006$	$0.835 \pm 0.011$	$0.835 \pm 0.010$	$0.703 \pm 0.006$
Identity	$0.804 \pm 0.009$	$0.798 \pm 0.015$	$0.795 \pm 0.017$	$0.656 \pm 0.006$
ReLU	$0.010 \pm 0.001$	$0.100 \pm 0.010$	$0.018 \pm 0.001$	$0.650 \pm 0.005$

$1 \times 10$ fold macro averaged results. Confidence interval of $2\sigma.$

The **UAF** is the activation function described in this paper. The non bold items are the other activation functions used for comparison.

To ensure fairness in the tests, all neurons and all layers are set to the same type of activation function. In the case of the UAF, a single UAF is applied to all neurons and to all layers. CIFAR-10 dataset contains 60,000 images in total, where 50,000 images are used for training and 10,000 images are used for testing. The training and testing datasets for CIFAR-10 are not randomized to allow comparisons between papers. After executing the $1 \times 10$ folding training and testing, the precision, recall, and $F_1$ scores of various activation functions are recorded in Table 2 . The ReLU activation function has the worst score $\mathrm{F}_1=0.018\pm 0.001$ because the ReLU’s gradient sometimes gets stuck and stops the weights from updating^19^. The identity, sigmoid, tanh, and ELU activation functions have poor scores $\mathrm{F}_1=0.795\pm 0.02$, $0.881\pm 0.006$, $0.835\pm 0.010$ and $0.886\pm 0.004$ because their gradients do not back-propagate well across many different CNN layers. On the other hand, Mish and LeakyReLU functions are designed to stop the gradient from reaching zero. As a result, they perform better and have higher scores $\mathrm{F}_1=0.891\pm 0.008$ and $0.893\pm 0.003$. Softplus and UAF have the highest scores $F_1 = 0.902$ due to the smoothness of the functions and being able to reach the global minimum. This means softplus and UAF are superior at classifying objects when compared to the other activation functions despite the UAF requires more training time for the UAF’s parameters to converge. Figure 2a shows the evolution of the UAF on the CIFAR-10 dataset. Upon initializing the UAF as the identity activation function, the UAF converges to a Mish activation function that is shifted to the right and has a different slope.

### Planetoid/CORA publication classification

In the Planetoid/CORA publication classification dataset^35^, uncategorized published papers and their publication metadata are given in order to classify the papers into one of seven academic fields. The input to the network is a graph of published papers, where each node contains the extracted keywords of a paper and each edge contains a citation between two papers. If a keyword exists within a paper, then it is labeled as 1, otherwise it is labeled as 0. The prediction uses a 64 layer graph convolutional neural (GCN) network^39^ that has 64 hidden channels and 1 dense layer. Bias weights are not used because they cause overfitting and performance degradation. To ensure fairness in the tests, all neurons and all layers are set to the same type of activation function. In the case of the UAF, a single UAF is applied to all neurons and to all layers. The Planetoid/CORA dataset contains 2708 publications in total. After randomly shuffling the dataset, 140 publications are randomly selected for training and 1000 publications are randomly selected for testing. Table 3 shows the $1 \times 10$ folding training and testing of the various activation functions. Sigmoid performs poorly $F_1 = 0.129 \pm 0.01$ due to label prediction imbalance. In the absence of bias weights, the Sigmoid skews the input domain of [0, 1] to the output range of [0.5, 0.731] and this leads to the overprediction of label 1 compared to the label 0. The same label prediction imbalance causes LogSigmoid, Hardswish, softplus, and SiLU to perform poorly. The ELU, identity, LeakyReLU, Mish, PReLU, ReLU, tanh, and UAF perform significantly better due to them not requiring bias weights. In Fig. 2b, the UAF converged to identity function and failed to obtain the best result because the ADAM optimizer stopped at a local minimum. Nevertheless, its $F_1$ score of $0.835\pm {0.008}$ is close to the best performed ReLU ($F_1=0.845 \pm 0.011$) which is able to preserve the information from the keywords.

**Table 3:** Planetoid/CORA classification using graph convolution neural networks.

Activation functions	Precision	Recall	$\mathrm{F}_1$	Training time (s)
ReLU	$0.845 \pm 0.013$	$0.852 \pm 0.011$	$0.845 \pm 0.011$	$47 \pm 6$
LeakyReLU	$0.843 \pm 0.010$	$0.852 \pm 0.003$	$0.844 \pm 0.004$	$44 \pm 1$
PReLU	$0.831 \pm 0.006$	$0.848 \pm 0.005$	$0.837 \pm 0.005$	$50 \pm 1$
ELU	$0.829 \pm 0.005$	$0.846 \pm 0.007$	$0.835 \pm 0.005$	$45 \pm 1$
**UAF**	$0.830 \pm 0.016$	$0.847 \pm 0.012$	$0.835 \pm 0.008$	$131 \pm 4$
Identity	$0.829 \pm 0.006$	$0.845 \pm 0.009$	$0.834 \pm 0.005$	$49 \pm 2$
Tanh	$0.826 \pm 0.004$	$0.846 \pm 0.005$	$0.833 \pm 0.003$	$45 \pm 2$
Mish	$0.742 \pm 0.015$	$0.740 \pm 0.022$	$0.737 \pm 0.005$	$49 \pm 1$
SiLU	$0.458 \pm 0.029$	$0.435 \pm 0.016$	$0.424 \pm 0.010$	$45 \pm 1$
Hardswish	$0.385 \pm 0.040$	$0.368 \pm 0.030$	$0.344 \pm 0.019$	$45 \pm 2$
Sigmoid	$0.124 \pm 0.023$	$0.163 \pm 0.006$	$0.129 \pm 0.011$	$45 \pm 1$
LogSigmoid	$0.105 \pm 0.026$	$0.152 \pm 0.013$	$0.112 \pm 0.019$	$46 \pm 2$
Softplus	$0.098 \pm 0.026$	$0.150 \pm 0.006$	$0.107 \pm 0.013$	$54 \pm 2$

$1 \times 10$ fold macro averaged results. Confidence interval of $2\sigma.$

The **UAF** is the activation function described in this paper. The non bold items are the other activation functions used for comparison.

### 9 gas quantification

The objective of the infrared spectra database is to predict the concentrations of 9 gasses using 1${\times }$1000 images of the gasses’ IR spectra. We generated the dataset using the similar procedure to^33^ and made the gas concentrations uniformly distributed between 0 and $10~\mathrm{\mu }M$. The total dataset contains 100,000 images. After shuffling, 80,000 images are randomly sampled for training and 20,000 images are randomly sampled for testing. A 2 layer MLP with 109 neurons each predicts the concentrations of the 9 gasses, of which the activation functions remain constant for all layers and all neurons. Table 4 shows the $1 \times 10$ fold testing of the 30 dB SNR IR spectra database^33^. The ReLU activation function again gets stuck and produces a high $\mathrm{RMSE}=1.2\pm 1.7$. Moreover, softplus, sigmoid, and tanh activation functions have high $\mathrm{RMSE}=0.90\pm 0.03$, $0.95\pm 0.01$ and $0.694\pm 0.002$ because they are not suited for quantification. On the other hand, MLPs using the Identity, LeakyReLU, and UAF activation functions obtained the lowest $\mathrm{RMSE}=0.489\pm 0.004$, $0.488\pm 0.004$ and $0.489\pm 0.003$ due to them being suitable for quantification. As a result, MLPs with the identity, LeakyReLU, and UAF are able to predict the concentrations of the gasses more accurately than the MLP with other activation functions. Fig. 2c shows the evolution of the UAF during the training procedure. The UAF begins as the identity function. Afterwards, the UAF changes to a parabolic function. Subsequently, the UAF converges to the identity function, which is close to the optimal activation function.

**Table 4:** 9 gas quantification using 2 layer MLP.

Activation functions	MAE ($\mathrm{\mu }$M)	RMSE ($\mathrm{\mu }$M)	Rel error	Training time ($\times 10^3$s)
PReLU	$0.271 \pm 0.002$	$0.487 \pm 0.003$	$0.3 \pm 0.2$	$2.77 \pm 0.17$
LeakyReLU	$0.269 \pm 0.002$	$0.488 \pm 0.004$	$0.3 \pm 0.1$	$2.87 \pm 0.16$
**UAF**	$0.269 \pm 0.001$	$0.489 \pm 0.003$	$0.3 \pm 0.1$	$2.93 \pm 0.11$
Identity	$0.273 \pm 0.002$	$0.489 \pm 0.004$	$0.4 \pm 0.1$	$2.84 \pm 0.18$
ELU	$0.273 \pm 0.001$	$0.490 \pm 0.003$	$0.4 \pm 0.1$	$2.87 \pm 0.16$
Mish	$0.333 \pm 0.005$	$0.527 \pm 0.004$	$0.8 \pm 0.5$	$2.90 \pm 0.13$
SiLU	$0.356 \pm 0.009$	$0.546 \pm 0.006$	$0.8 \pm 0.5$	$2.91 \pm 0.21$
Hardswish	$0.365 \pm 0.096$	$0.556 \pm 0.005$	$0.7 \pm 0.1$	$2.91 \pm 0.17$
Tanh	$0.521 \pm 0.002$	$0.694 \pm 0.002$	$0.9 \pm 0.4$	$2.89 \pm 0.11$
Softplus	$0.647 \pm 0.034$	$0.900 \pm 0.059$	$2.3 \pm 0.8$	$2.84 \pm 0.16$
Sigmoid	$0.564 \pm 0.009$	$0.953 \pm 0.013$	$1.7 \pm 0.9$	$2.81 \pm 0.14$
ReLU	$1.146 \pm 1.156$	$1.231 \pm 1.725$	$0.4 \pm 0.2$	$2.73 \pm 0.05$
LogSigmoid	$4.996 \pm 0.012$	$5.771 \pm 0.012$	$1.00 \pm 0.01$	$2.99 \pm 0.22$

Infrared spectra database for 9 gas quantification^33^ 30 dB SNR uniformly distributed concentrations.

$1 \times 10$ fold macro averaged results. Confidence interval of $2\sigma.$

The **UAF** is the activation function described in this paper. The non bold items are the other activation functions used for comparison.

### ZINC molecular solubility quantification

The objective of the ZINC molecular solubility quantification dataset^36^ is to predict an unknown chemical’s solubility property given its molecular structure. A graph neural network with principal neighbourhood aggregation^40^ is used to predict the solubility values. For testing, a single type of activation function is applied to all layers and all neurons. The input to the neural network is the molecular structure in the form of a graph. Each node represents an atom and each edge represents a bond between two atoms. The entire ZINC dataset contains 250,000 different molecular graphs. 220,011 molecular graphs are randomly sampled for training and 5,000 molecular graphs are randomly sampled for testing. Table 5 shows the results of the various activation functions on the ZINC dataset after executing the $$1 \times 10$$ fold testing. Sigmoid and LogSigmoid perform poorly $$RMSE = $$ $$0.6 \pm 0.1$$ and $$0.51 \pm 0.05$$ because they are not designed for quantification. Identity performs poorly $$RMSE = 0.56 \pm 0.05$$ as it does not filter out invalid values such as negative solubility values. Softplus, Tanh, ELU, ReLU, PReLU, and LeakyReLU perform moderately well but they do not achieve the best performance. This is because the output probability distributions of the activation functions above do not match the actual probability distribution of the ZINC dataset. On the other hand, UAF, Hardswish, Mish, and SiLU obtained better performances $$RMSE = 0.47 \pm 0.04$$, $$0.46 \pm 0.08$$, $$0.48 \pm 0.04$$, $$0.47 \pm 0.05$$ because they are able to approximate the probability distribution of the ZINC dataset with greater accuracy. The confidence interval of the activation function with the lowest RMSE, Hardswish, overlaps significantly with the confidence interval of UAF, Mish, and SiLU. As a result, it is unknown which activation function is optimal for this specific problem.

**Table 5:** ZINC molecular solubility quantification using graph neural networks with principal neighbourhood aggregation.

Activation function	MAE	RMSE	Rel error	Training time ($\times 10^3$s)
Hardswish	$0.16 \pm 0.01$	$0.46 \pm 0.08$	$0.46 \pm 0.11$	$2.24 \pm 0.02$
**UAF**	$0.17 \pm 0.01$	$0.47 \pm 0.04$	$0.50 \pm 0.09$	$4.62 \pm 0.04$
SiLU	$0.17 \pm 0.01$	$0.47 \pm 0.05$	$0.45 \pm 0.07$	$2.27 \pm 0.01$
ReLU	$0.18 \pm 0.01$	$0.48 \pm 0.03$	$0.51 \pm 0.14$	$2.16 \pm 0.01$
Mish	$0.17 \pm 0.02$	$0.48 \pm 0.04$	$0.44 \pm 0.06$	$2.25 \pm 0.06$
ELU	$0.18 \pm 0.01$	$0.49 \pm 0.04$	$0.47 \pm 0.08$	$2.24 \pm 0.01$
LeakyReLU	$0.18 \pm 0.01$	$0.49 \pm 0.04$	$0.54 \pm 0.09$	$2.17 \pm 0.06$
PReLU	$0.190 \pm 0.008$	$0.51 \pm 0.04$	$0.48 \pm 0.07$	$2.17 \pm 0.02$
Softplus	$0.19 \pm 0.01$	$0.51 \pm 0.04$	$0.52 \pm 0.09$	$2.27 \pm 0.01$
LogSigmoid	$0.20 \pm 0.02$	$0.51 \pm 0.05$	$0.53 \pm 0.11$	$2.27 \pm 0.03$
Tanh	$0.19 \pm 0.02$	$0.51 \pm 0.06$	$0.53 \pm 0.06$	$2.26 \pm 0.01$
Identity	$0.25 \pm 0.01$	$0.56 \pm 0.05$	$0.73 \pm 0.13$	$2.24 \pm 0.01$
Sigmoid	$0.27 \pm 0.04$	$0.61 \pm 0.11$	$0.72 \pm 0.14$	$2.25 \pm 0.02$

$1 \times 10$ fold macro averaged results. Confidence interval of $2\sigma.$

The **UAF** is the activation function described in this paper. The non bold items are the other activation functions used for comparison.

### BipedalWalker-v2 reinforcement learning

The goal of the BipedalWalker-v2^34^ RL benchmark is to move the robot past the finish line while adapting to large changes in the simulation’s terrain. The neural networks control the torques of the robot’s legs in order to move the robot forwards and to prevent the robot from falling over. The reward function depends on the furthest distance traveled and the total amount of energy used to move the robot. Maximizing the furthest distance traveled and minimizing the total energy used, increases the reward function. Moreover, the neural networks must converge in the least number of epochs. High rewards and low number of epochs imply that the models run efficiently. Table 6 shows the results of the Deep Deterministic Policy Gradient^41^ on BipedalWalker-v2. $1 \times 10$ fold testing is used on the dataset and each fold has randomly generated terrain. The sigmoid activation function achieves the 100 reward in $818 \pm 213$ epochs, which is the least number of epochs. UAF is slightly slower at achieving the 100 reward in $859 \pm 209$ epochs. However, UAF is the fastest at achieving the 250 reward with $961 \pm 193$ epochs. In the long run, the UAF achieves the best performance in terms of highest rewards in the least number of epochs.

**Table 6:** BipedalWalker-v2 using deep deterministic policy gradient.

Activation functions	100 reward ($\times 10^3$ Epochs)	250 reward ($\times 10^3$ Epochs)	40 distance ($\times 10^3$ Epochs)	88 distance ($\times 10^3$Epochs)	Training time ($\times 10^3$s)
**UAF**	$0.86 \pm 0.21$	$0.96 \pm 0.19$	$0.77 \pm 0.13$	$0.83 \pm 0.19$	$6.98 \pm 2.38$
LeakyReLU	$0.85 \pm 0.16$	$0.99 \pm 0.16$	$0.77 \pm 0.14$	$0.82 \pm 0.15$	$6.14 \pm 1.72$
ReLU	$0.89 \pm 0.16$	$0.99 \pm 0.19$	$0.80 \pm 0.17$	$0.83 \pm 0.18$	$5.53 \pm 0.86$
Softplus	$0.84 \pm 0.53$	$0.99 \pm 0.59$	$0.68 \pm 0.33$	$0.75 \pm 0.29$	$4.94 \pm 2.69$
Sigmoid	$0.82 \pm 0.21$	$1.00 \pm 0.41$	$0.72 \pm 0.23$	$0.79 \pm 0.27$	$5.93 \pm 1.98$
ELU	$0.93 \pm 0.16$	$1.17 \pm 0.45$	$0.82 \pm 0.22$	$0.87 \pm 0.13$	$5.86 \pm 2.05$
Tanh	$0.92 \pm 0.20$	$1.22 \pm 0.48$	$0.83 \pm 0.15$	$0.90 \pm 0.20$	$4.46 \pm 2.27$
Mish	$0.99 \pm 0.23$	$1.29 \pm 0.25$	$0.89 \pm 0.12$	$0.94 \pm 0.13$	$6.95 \pm 2.02$
Identity	Not reached	Not reached	$1.38 \pm 0.36$	$1.49 \pm 0.05$	$2.50 \pm 1.29$

$1 \times 10$ fold macro averaged results. Confidence interval of $2\sigma.$

Figure 2e shows the evolution of the UAF in BipedalWalker-v2. The UAF is initialized as the identity function. Subsequently, the UAF evolves to an unusual parabolic activation function. The parabolic function is a new activation function that performs well for this specific problem. It limits the torque of the bipedal robot to $y \in [-1,\infty )$ and the parabolic function decreases the energy needed to move the robot. As the energy needed decreases, the reward increases.

## Conclusion and future work

The UAF was developed as a generic activation function that can approximate many others such as the identity, ReLU, LeakyReLU, sigmoid, tanh, softplus, and Gaussian as well as to evolve to a unique shape. This versatility allows the UAF to achieve near optimal performance in classification, quantification, and reinforcement learning. As demonstrated, incorporating the UAF in a neural network leads to best or close-to-best performance, without the need to try many different activation functions in the design.

In the current setup, a single UAF is applied to the entire neural network. As for future work, each layer or each neuron could have its own UAF. Each UAF would then specialize to a specific task. This would enable the neural networks to model more non-linear processes and to solve more difficult problems. Moreover, the UAF could be used for transfer learning. The activation functions from one neural network could be transferred to another neural network. This would enable multiple neural networks to learn from each other and to converge faster.

## Supplementary Information

Supplementary Information.

Supplementary Video 1.

Supplementary Video 2.

## Data Availability

The majority of the datasets used in this paper are publicly available. Private datasets can be given upon request.

## Code availability

The UAF’s code is available for Tensorflow and Pytorch upon request.
