# Supplementary material for: Universal activation function for machine learning
Source: Sci Rep. 2021 Sep 21;11:18757. doi: 10.1038/s41598-021-96723-8 (PMC8455573; doi:10.1038/s41598-021-96723-8)
Supplement: Supplementary file 1 — Supplementary Information. [file 41598_2021_96723_MOESM1_ESM.pdf]

Supplementary Information
Universal Activation Function For Machine Learning

Brosnan Yuen, Minh Tu Hoang, Xiaodai Dong, and Tao Lu

S.1. Error Analysis of UAF

In this section, the UAF's approximations of the various common activation functions will be described.

A. Identity Function

By setting $A = 1$, $B = 0$, $C = 0$, $D = -1$, and $E = 0$, the $f_{UAF}(x)$ becomes

$$f_{UAF}(x) = \ln\left(\frac{1+e^{x}}{1+e^{-x}}\right) = x \tag{S1}$$

which is identical to the identity function.

B. Step Function

If $A = D \to \infty$, $B = \frac{1}{2A}$, $C = E = 0$, then the $f_{UAF}(x)$ becomes

$$f_{UAF}(x) = \lim_{A\to\infty} \ln\left(\frac{1+e^{Ax+0.5}}{1+e^{Ax-0.5}}\right) = \begin{cases} 1 & \text{if } x > 0 \\ 0.5 & \text{if } x = 0 \\ 0 & \text{otherwise} \end{cases} \tag{S2}$$

which is identical to the step function $f_{step}(x)$. In practical implementations, A is set to a finite large number that does not cause overflows. The error $\mathcal{E}$ between the $f_{UAF}(x)$ and the targeted function $f_{act}(x)$ (in this section, $f_{act}(x) = f_{step}(x)$)

$$\mathcal{E} = f_{UAF}(x) - f_{act}(x) \tag{S3}$$

can be analyzed as follows. At $x = 0$, the $f_{UAF}(0) = \ln\left(\frac{1+e^{0.5}}{1+e^{-0.5}}\right) = 0.5$ and this matches step function's value. For values $Ax \gg 1$, $f_{UAF}(x) \approx 1.0$ as $e^{Ax\pm0.5} \gg 1$ while for $Ax \ll -1$, $f_{UAF}(x) \approx 0$ since $e^{Ax\pm0.5} \ll 1$. The derivative of the $f_{UAF}(x)$ is

$$f'_{UAF}(x) = \frac{Ae^{Ax}(e^{0.5} - e^{-0.5})}{(1+e^{Ax-0.5})(1+e^{Ax+0.5})} > 0, \forall x \in \mathbb{R} \tag{S4}$$

Therefore, for $x > 0$, the error $\mathcal{E}(x) < 0$ suggesting the absolute error $|\mathcal{E}|$ monotonically decreases as x increases in this regime. For $x < 0$, $\mathcal{E}(x) > 0$ and $|\mathcal{E}|$ montontonically increases as x increases. The maximum absolute errors occur when x approaches zero from both sides. Due to the discontinuity of the step function, the error reaches ±0.5 as $x \to 0^{\pm}$.

To quantitatively analyze the error between the UAF and the step function, we plot them for $x \in (-\frac{10}{A}, \frac{10}{A})$. Here, $A = D = 70.9992$, $B = 0.007042$, and $E = C = 0$. As shown in Fig. 1a), the solid black line represents the $f_{UAF}(x)$, the dashed green line represents the step function, and the solid red line represents the error. Near $x = 0$, the error is very high $|\mathcal{E}| = 0.5$. As x moves away from $x = 0$, the error decreases to $|\mathcal{E}| = 5 \times 10^{-5}$ at $x = \pm\frac{10}{A}$. As a result, the absolute errors for points out of range are smaller than 5×10^{-5}. Clearly our UAF well approximates the step function for $x \in (-\infty, \infty)$.

C. Sigmoid Function

As the step function is similar to the sigmoid function, the step function transforms into the sigmoid function, when $A = D \approx 1.01605291$. The optimal value of A is found using a gradient descent optimizer. The other parameters are setup exactly the same way as in the step function case with $B = \frac{1}{2A} = 0.492100$, $C = E = 0$, written as

$$f_{UAF}(x) = \ln\left(\frac{1+e^{Ax+0.5}}{1+e^{Ax-0.5}}\right) \tag{S5}$$

and it gives a good approximation of the sigmoid function $\sigma(x)$ given by

$$\sigma(x) = \frac{1}{1+e^{-x}}. \tag{S6}$$

The UAF approximation is bounded by the maximum absolute errors. In order to find the maximum absolute errors, the derivative of the error is set to zero $\frac{\mathrm{d}\mathcal{E}}{\mathrm{d}x} = 0$ and the characteristic equation

$$(e-1)A(e^{x}+1)^2e^{Ax-0.5} + e^{x}(e^{Ax-0.5}+1)(-e^{Ax+0.5}-1) = 0 \tag{S7}$$

is solved to get the critical points located at $x \approx \pm0.866499$. The points have a maximum absolute error of

$$|\mathcal{E}| = \ln\left(\frac{1+e^{A(0.866499)+0.5}}{1+e^{(0.866499)-0.5}}\right) - \frac{1}{1+e^{-0.866499}} \approx 0.000616 \tag{S8}$$

for the approximation.

Similar to the case of the step function discussed above, at $x = 0$, the $f_{UAF}(0) = \ln\left(\frac{1+e^{0.5}}{1+e^{-0.5}}\right) = 0.5$ and this matches sigmoid function's value. As x increases past zero, the absolute error increases up until the maximum absolute error $|\mathcal{E}| \approx 0.000616$ at $x \approx 0.866499$. As x increases past that point, the absolute error monotonically decreases because the derivative is always positive $f'_{UAF}(x) > 0$ as mentioned in (S4) and the upper bound of the function $f_{UAF}(x) = \sigma(x) \approx 1$, $\forall Ax \gg 1$. The inverse is also true. As x decreases past the maximum error $x \approx -0.866499$, the error monotonically decreases because the derivative is always positive $f'_{UAF}(x) > 0$ and the function has a lower bound $f_{UAF}(x) = \sigma(x) \approx 0$, $\forall Ax \ll -1$. As shown in Fig. 1b), the solid black line represents the $f_{UAF}(x)$, the dashed green line represents the sigmoid function, and the solid red line represents the error. Near $Ax = 1$, the absolute error is

high $|\mathcal{E}| = 6 \times 10^{-4}$. As x moves away from the maximum absolute errors, the absolute error decreases to $|\mathcal{E}| = 6 \times 10^{-6}$ at $x = \pm\frac{10}{A}$. This means the absolute error will always be less than $|\mathcal{E}| = 6 \times 10^{-6}$ for values $x > \frac{10}{A}$ or equivalently $x < -\frac{10}{A}$.

D. Tanh Function

The tanh function is similar to the sigmoid function. By setting $B = \frac{1}{A}$, $C = 0$, $D = A$, $E = -1$ and optimizing the parameter A, the sigmoid function transforms into the tanh function. Parameter B is twice as large because tanh has twice the range of sigmoid. For the best fit, set $A \approx 2.12616013$ and the UAF becomes

$$f_{UAF}(x) = \ln\left(\frac{1+e^{Ax+1}}{1+e^{Ax-1}}\right) - 1, \tag{S9}$$

an approximation of the $\tanh(x)$ function given by

$$\tanh(x) = \frac{e^x - e^{-x}}{e^x + e^{-x}}. \tag{S10}$$

The UAF approximation is bounded by the maximum absolute errors. In order to find the maximum absolute errors, the derivative of the error is set to zero $\frac{\mathrm{d}\mathcal{E}}{\mathrm{d}x} = 0$ and it leads to a characteristic equation given by

$$\begin{aligned} &- Ae^{Ax-1} + Ae^{Ax+1} - 2Ae^{Ax+2x-1} + 2Ae^{Ax+2x+1} \\ &- Ae^{Ax+4x-1} + Ae^{Ax+4x+1} - 4e^{Ax+2x-1} - 4e^{Ax+2x+1} \\ &\qquad - 4e^{2Ax+2x} - 4e^{2x} = 0. \end{aligned} \tag{S11}$$

Numerically solving the above equation, we found four roots at $x = \pm 0.435499$, $x = \pm 3.4355$. Among them, the maximum absolute error $|\mathcal{E}| \approx 0.004719$ occurs at $x \approx \pm 0.435499$. Note that for $x > 3.4355$, the error derivative has no more zero crossing points, suggesting the error will monotonically decrease to zero from $\mathcal{E} = 0.00383$ at $x = 3.4355$. Similarly, for $x < -3.4355$, the error will monotonically approach zero from $\mathcal{E} = -0.00383$ at $x = -3.4355$. The change of error as a function of x is clearly demonstrated as red solid line in Fig. 1c). As shown, $|\mathcal{E}| = 6 \times 10^{-5}$ at $x = \pm\frac{10}{A}$. This means the absolute error will always be less than $|\mathcal{E}| = 6 \times 10^{-5}$ for values $x > \frac{10}{A}$ or equivalently $x < -\frac{10}{A}$.

E. ReLU Function

If $B = C = E = 0$, $D = A - 1$, $A \to \infty$, the $f_{UAF}(x)$ becomes

$$\begin{aligned} f_{UAF}(x) &= \lim_{A\to\infty} \ln\left(\frac{1+e^{Ax}}{1+e^{Ax-x}}\right) \\ &= \begin{cases} x, & x \geq 0 \\ 0, & x < 0 \end{cases} \end{aligned} \tag{S12}$$

which is identical to the $ReLU(x)$ function.

In practical implementations, A is set to a large finite number that avoids overflows. Under such circumstances, the maximum absolute errors occur where the derivative of error becomes zero. For positive interval $x > 0$, this leads to a characteristic equation

$$(A-1)e^{Ax} - Ae^{(A-1)x} - 1 = 0. \tag{S13}$$

The maximum absolute error occurs at position $x \approx \ln\left(\frac{A}{A-1}\right) \approx 0.0181$ and the value of the maximum absolute error is

$$|\mathcal{E}| \approx \left|\ln\left(\frac{1+(\frac{A}{A-1})^A}{1+(\frac{A}{A-1})^{A-1}}\right) - x\right| \approx 0.00395. \tag{S14}$$

For the negative interval $x < 0$, this leads to a characteristic equation

$$Ae^x + e^{Ax} + 1 = A. \tag{S15}$$

The maximum absolute error occurs at position $x \approx \ln\left(\frac{A-1}{A}\right) \approx -0.0181$ and the value of the maximum absolute error is

$$|\mathcal{E}| \approx \left|\ln\left(\frac{1+(\frac{A-1}{A})^A}{1+(\frac{A-1}{A})^{A-1}}\right)\right| \approx 0.00395. \tag{S16}$$

At $x = 0$, the $f_{UAF}(0) = \ln\left(\frac{1+e^0}{1+e^0}\right) = 0$ and this matches ReLU function's value. As x increases past zero, the absolute error increases up until the maximum absolute error $|\mathcal{E}| \approx 0.00395$ at $x \approx 0.0181$. As x increases past that point, the absolute error monotonically decreases and converges to zero. For the negative interval $x < 0$, as x decreases past the maximum absolute error point $x \approx -0.0181$, the absolute error monotonically decreases and converges to zero. Fig. 1d) shows a red line that represents the error of the UAF. Around the neighbourhood $x = 0$, the error is high. As x moves away from the neighbourhood around $x = 0$, the error decreases to $|\mathcal{E}| = 7 \times 10^{-6}$ at $x = \pm\frac{10}{A}$. This means the absolute error will always be less than $|\mathcal{E}| = 7 \times 10^{-6}$ for values $x > \frac{10}{A}$ or equivalently $x < -\frac{10}{A}$.

F. LeakyReLU Function

LeakyReLU functions have a parameter $\alpha \in (0, 0.1]$ that controls the negative slope. By setting the parameters $A = 1$, $B = C = E = 0$, $D = -\alpha$,

$$f_{UAF}(x) = \ln\left(\frac{1+e^x}{1+e^{-\alpha x}}\right) \tag{S17}$$

gives an approximation of the LeakyReLU function

$$LeakyReLU(x) = \begin{cases} x, & x \geq 0 \\ \alpha x, & x < 0 \end{cases} \tag{S18}$$

The maximum absolute errors occur where the derivative of error $\frac{\mathrm{d}\mathcal{E}}{\mathrm{d}x} = 0$. For positive interval $x > 0$, this leads to a characteristic equation

$$(\alpha - 1)e^{-\alpha x} + \alpha e^{x-\alpha x} - 1 = 0. \tag{S19}$$

For negative interval $x < 0$, this leads to a characteristic equation.

$$\alpha(-e^x - 1) + e^x(e^{-\alpha x} + 1) = 0. \tag{S20}$$

If $\alpha = 0.1$, the maximum absolute error is $|\mathcal{E}| = 0.5050$ at position $x = \pm 3.106$. At $x = 0$, $f_{UAF}(0) = \ln\left(\frac{1+e^0}{1+e^0}\right) =$

0 and this matches the LeakyReLU's value. As x decreases below 0, the absolute error increases up until the maximum absolute error. After decreasing past the point $x = -3.106$, the absolute error given by

$$\mathcal{E}(x) = \ln\left(\frac{1+e^{x}}{1+e^{-\alpha x}}\right) - \alpha x = \ln\left(\frac{1+e^{x}}{1+e^{\alpha x}}\right) \tag{S21}$$

monotonically decreases and converges to zero $\lim_{x\to-\infty}\mathcal{E}(x) = 0$. The inverse is also true. As x increases above 0, the absolute error increases up until the maximum absolute error. After increasing past the point $x = 3.106$, the absolute error given by

$$\mathcal{E}(x) = \ln\left(\frac{1+e^{x}}{1+e^{-\alpha x}}\right) - x = \ln\left(\frac{1+e^{-x}}{1+e^{-\alpha x}}\right) \tag{S22}$$

monotonically decreases and converges to zero $\lim_{x\to\infty}\mathcal{E}(x) = 0$. Fig. 1e) represents the error of the UAF as a red line. Around the neighbourhood $x = 0$, the error is very high $|\mathcal{E}| = 0.505$. As x moves away from $x = 0$, the error decreases to $|\mathcal{E}| = 0.00677$ at $x = \pm\frac{50}{A}$. This means the absolute error will always be less than $|\mathcal{E}| = 0.00677$ for values $x > \frac{50}{A}$ or equivalently $x < -\frac{50}{A}$.

G. Softplus Function

If $A = 1$, $B = 0$, $C = 0$, $D = 0$ and $E = \ln(2)$, the $f_{UAF}(x)$ becomes

$$\begin{aligned} f_{UAF}(x) &= \ln\left(\frac{1+e^{1(x+0)+0x^2}}{1+e^{0(x-0)}}\right) + \ln(2) \\ &= \ln(1+e^{x}) \end{aligned} \tag{S23}$$

which exactly equals the softplus function because the UAF is built using the softplus function.

H. Gaussian Function

A gradient descent optimizer is used to find the parameters $C = -0.61341425$ and $E = \ln(2)$ such that the UAF with the form

$$f_{UAF}(x) = \ln(1+e^{-0.61341425x^2}) \tag{S24}$$

approximates a scaled version of the Gaussian function $H(x)$

$$H(x) = \ln(2)e^{-\frac{x^2}{2}} \tag{S25}$$

with minimum RMSE over $x \in (-\infty, \infty)$. The other parameters are not needed and they are set to zero $A = B = D = 0$. The maximum absolute error occurs when the derivative of the absolute error is zero, which leads to the characteristic equation

$$\frac{2Cxe^{Cx^2}}{1+e^{Cx^2}} + x\ln(2)e^{-\frac{x^2}{2}} = 0 \tag{S26}$$

and the critical points occur at $x \approx \pm 0.8821$ and has a value of $|\mathcal{E}| = 0.0129$

At $x = 0$, the $f_{UAF}(0) = \ln\left(1+e^{0}\right) = \ln(2)$ and this matches Gaussian function's value $N(0) = \ln(2)$. As x increases past zero, the absolute error increases up until the maximum absolute error $|\mathcal{E}| \approx 0.0129$ at $x \approx 0.8821$. As x increases past that point $-Cx \gg 4$, the absolute error converges to zero because the UAF goes to zero $f_{UAF}(x) \approx 0$ and the Gaussian function goes to zero $N(x) \approx 0$. The inverse is also true. Fig. 1f) shows a red line that represents the error of the UAF. Around the neighbourhood $x = 0$, the error is high $|\mathcal{E}| = 0.0129$. As x moves away from $x = 0$, the absolute error exponentially decreases to $|\mathcal{E}| = 5 \times 10^{-10}$ at $x = \pm\frac{4}{C}$. This means the absolute error will always be less than $|\mathcal{E}| = 5 \times 10^{-10}$ for values $x > -\frac{4}{C}$ or equivalently $x < \frac{4}{C}$.

S.2. Implementation Details

Batch normalization $BN(x)$ is widely used by researchers

$$BN(x) = \frac{x - \mu_x}{\sigma_x} \tag{S27}$$

where μ_x, σ_x are the mean and the standard deviation of input x respectively. Batch normalization is normally applied to the data before the activation function. This effectively reduces the input x domain of UAF to $x \in [-100, 100]$, which reduces floating point overflows. The softplus function [1] can be rewritten to

$$log1p(x) = \log(1+x) \tag{S28}$$

$$\ln(1+e^{x}) = ReLU(x) + log1p(e^{-|x|}) \tag{S29}$$

in order to increase the floating point precision. The UAF can be rewritten to

$$\begin{aligned} f_{UAF}(x) = &\ ReLU(A(x+B)+Cx^2) \\ &+log1p(e^{-|A(x+B)+Cx^2|}) \\ &+ReLU(D(x-B)) \\ &+log1p(e^{-|D(x-B)|}) \\ &+E \end{aligned} \tag{S30}$$

and this minimizes the floating point overflow errors.

References

[1] M. Abadi et al., "TensorFlow: Large-scale machine learning on heterogeneous systems," 2015, software available from tensorflow.org. [Online]. Available: https://www.tensorflow.org/
